# A Treatise on the Diseases of the Nervous System

**Published:** 1876-08

**Authors:** 


A Treatise on the Diseases of the Nervous System. By William A. Hammond, Professor of the Diseases of the Mind and Nervous System in the Medical Department of the University of the city of New York, etc. Sixth Edition. New York: D. Appleton & Co., 549 and 551 Broadway. 1876. For sale by Burke & Hancock, 26 Alabama Street, Atlanta, Ga.

This work contains five sections, treating respectively of the diseases of the brain, of the spinal cord, of the cerebro-spinal system, of the peripheral system of nerves, and of toxic diseases of the nervous system. It is in our judgment one of the best works in American medical literature on this subject, for the information and guidance of the medical practitioner in every-day practice; because, without being too voluminous, it contains everything of importance for a correct conception of the pathology and treatment of this class of diseases, as far as the present status of our knowledge in this part of medical science enables any writer to embrace this information into one work.

The opinions of the author, the result of his own extensive practical experience in the treatment of these diseases, form one of the most valuable parts of this work.

The numerous illustrations are well executed, are strikingly demonstrative, and add much to the instructive value of the text.

We know of no work on this important branch of medical knowledge of greater value than the one alluded to in this notice, and therefore recommend it heartily to our medical friends and patrons.

